# Omega-3 Polyunsaturated Fatty Acids: Benefits and Endpoints in Sport

**DOI:** 10.3390/nu11010046

**Published:** 2018-12-27

**Authors:** Maria Alessandra Gammone, Graziano Riccioni, Gaspare Parrinello, Nicolantonio D’Orazio

**Affiliations:** 1 Human and Clinical Nutrition Unit, Department of Medical, Oral and Biotechnological Sciences, University G. D’Annunzio, 66100 Chieti, Italy; griccioni@gmail.com (G.R.); ndorazio@unich.it (N.D.); 2 Cardiology Unit, Cardiology Department, San Camillo De Lellis Hospital, Manfredonia, 71100 Foggia, Italy; 3 Department of Internal and Specialistic Medicine DIBIMIS, University of Palermo, 90123 Palermo, Italy; gaspare.parrinello@unipa.it

**Keywords:** PUFAs, omega-3, marine bioactives, sport, inflammation, seafood, functional foods, nutrition

## Abstract

The influence of nutrition has the potential to substantially affect physical function and body metabolism. Particular attention has been focused on omega-3 polyunsaturated fatty acids (*n*-3 PUFAs), which can be found both in terrestrial features and in the marine world. They are responsible for numerous cellular functions, such as signaling, cell membrane fluidity, and structural maintenance. They also regulate the nervous system, blood pressure, hematic clotting, glucose tolerance, and inflammatory processes, which may be useful in all inflammatory conditions. Animal models and cell-based models show that *n*-3 PUFAs can influence skeletal muscle metabolism. Furthermore, recent human studies demonstrate that they can influence not only the exercise and the metabolic response of skeletal muscle, but also the functional response for a period of exercise training. In addition, their potential anti-inflammatory and antioxidant activity may provide health benefits and performance improvement especially in those who practice physical activity, due to their increased reactive oxygen production. This review highlights the importance of n-3 PUFAs in our diet, which focuses on their potential healthy effects in sport.

## 1. Introduction

There is an increasing interest in finding nutrients and supplements that can improve athletic performance and recovery. Athletes often use dietary supplements in order to increase metabolic capacity, delay fatigue onset, improve muscle hypertrophy, and shorten recovery periods. In addition, athletes can often face a reduction in immune function, due to intense exercise training and a frequent challenging competition, which becomes more prone to upper respiratory tract infections. Moreover, exercise training exerts a physiologic stress on the body, which requires a coordinated response by the cardiovascular, pulmonary, and nervous systems to increase the blood flow and the oxygen supply to the working skeletal muscle. At rest, muscle receives approximately 20% of the total blood flow, but, during exercise, this can increase to more than 80%.

Ergogenic aids may help prepare an individual to exercise, improve exercise efficiency, enhance recovery from exercise, or assist in injury prevention during intense training. In this respect, omega-3 have been recently considered as an ergogenic supplement, which may have a role in these processes, which not only contrast exercise-induced inflammation, but also improve the health of muscle and its energy availability [1].

Omega-3 are polyunsaturated fatty acids (PUFAs) with more than one carbon-carbon double bond in their backbone. They are polyunsaturated because their chain comprises several double bonds. One way in which a fatty acid is named is determined by the location of the first double bond, counted from the tail, that is, the omega (ω-) or the n- end. Thus, in omega-3 fatty acids, the first double bond is between the third and fourth carbon atoms from the tail end. These essential nutrients have to be introduced through diet. They can be found in fish such as sardines, salmon, tuna, halibut, and other seafood such as algae and krill [2], and in lake trout, in some plants, and nut oils. These PUFAs, which are stored in membrane phospholipids, are responsible for numerous cellular functions including the maintenance of the cell membrane structure, fluidity, signaling, and cell-to-cell interaction. 

*N*-3 PUFAs can reduce inflammation and may help lower risk of chronic diseases such as heart disease, cancer, and arthritis. They also regulate blood pressure, hematic clotting, glucose tolerance, and nervous system development and functions [3]. Among omega-3, there are α-linolenic acid (ALA), eicosapentaenoic acid (EPA), and docosahexaenoic acid (DHA). Omega-3 fatty acids are also named “vitamin F” from “fatty acids” [4]. EPA and DHA are found in cold water fishes, which possess a greater quantity of body fat, although their content in EPA and DHA depends on some variables such as climate, the environment, and a fish diet [5]. ALA is found in flaxseeds, canola (rapeseed) oil, soybeans, pumpkin seeds, perilla seed oil, walnuts, and their derivative oils. Healthy effects come mostly from EPA and DHA. ALA from flax and other vegetarian sources needs to be converted in the body to EPA and DHA. Other important marine sources of *n*-3 PUFAs include sea life such as krill, algae, microalgae, and crustaceans. Krill oil, in particular Antarctic krill, is a rich source of both antioxidants, such as marine carotenoids (for example astaxanthin and fucoxanthin), vitamins A and E, and phospholipids containing long-chain *n*-3 PUFAs. In fact, alternative EPA and DHA marine sources such as sponges, bacteria, fungi, plants, and, in particular, autotrophic macroalgae and microalgae, are currently being explored for large-scale commercial omega-3 production [6] because of their optimum balance between *n*-3 and *n*-6 fatty acids [7]. In particular, brown and red algae are characterized by the presence of EPA and ALA [8] as well as green seaweeds, such as *Ulva pertusa*, which are rich in hexadecatetraenoic acid [9], and octadecatetraenoic acid, which is abundant in *Laminaria* sp. and *Undaria pinnatifida* [10].

## 2. Exercise-Induced Oxidative Stress and Inflammation: The Paradox of Intense Sport Exercise

**Figure 1:**
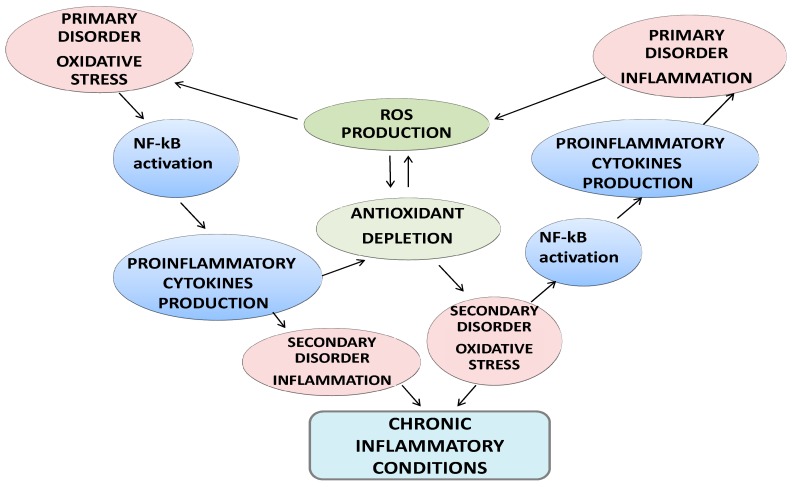
Crosstalk between oxidative stress and inflammation and clinical consequences.

Oxidative stress is usually defined as a disturbance in the pro-oxidant-antioxidant balance in favor of the former. During periods of oxidative stress, pro-oxidants overwhelm the antioxidant defenses in cells and damage cellular constituents [11]. Thus, oxidative stress in biological systems is often characterized by an increase in the formation of free radicals and other oxidants, a decrease in small-molecular-weight and/or lipid-soluble antioxidants, a disturbance in cellular redox balance, and oxidative damage to cellular components (i.e., lipids, proteins, and/or DNA). If an atom/molecule contains one or more unpaired electrons and is capable of independent existence, it is referred to as a “free radical” [11]. Free radicals can be generated as products of homolytic, heterolytic, or redox reactions, which produce either charged or uncharged radical species. Reactive oxygen species (ROS) is a general term that refers to not only oxygen-centered radicals but also includes nonradical but reactive derivatives of oxygen (e.g., hydrogen peroxide). Similarly, the term reactive nitrogen species (RNS) refers to both nitrogen radicals along with other reactive molecules in which the reactive center is nitrogen. The primary free radicals generated in cells are superoxide (O_2_-) and nitric oxide (NO). Superoxide is either generated through an incomplete reduction of oxygen in electron transport systems or as a specific product of enzymatic systems, while NO is generated by a series of specific enzymes (the nitric oxide synthases). Both superoxide and NO are reactive and can readily react to form a series of other ROS and RNS [11]. Free radicals can be produced in all cellular compartments and, ultimately, results in protein damage [12]. Furthermore, the exposure of biological systems to various conditions of oxidative stress leads to age-dependent increases in the cellular levels of oxidatively modified proteins, lipids, and nucleic acids, and subsequently predisposes the object to the development of age-related disorders. Because oxidative protein folding occurs in the endoplasmic reticulum and perturbations in protein folding can cause deleterious consequences, alterations in redox status or generation of ROS/RNS could directly and indirectly affect endoplasmic homeostasis and protein folding [12]. There is a close interdependence between oxidative stress and inflammation (Figure 1). When oxidative stress appears, inflammation develops as a secondary disorder and further enhances oxidative stress. On the other hand, inflammation can induce oxidative stress as a secondary disorder, which can further enhance inflammation [13]. At the site of inflammation, the activated inflammatory cells release many enzymes (such as neutral proteases, elastase, collagenase, acid hydrolases, phosphatases, and lipases), reactive species (superoxide, hydrogen peroxide, hydroxyl radical, and hypochlorous acid), and chemical mediators (eicosanoids, complement components, cytokines, chemokines, and nitric oxide) and, thereby, induce tissue damage and further oxidative stress [13]. 

Besides many health benefits, paradoxically, intense exercise can result in oxidative damage to cellular constituents. The skeletal muscle usually produces free radicals, which are unstable molecules oxidizing other molecules in order to become stable, in basal conditions. This production increases during contractile activity. In fact, aerobic exercise augments oxygen consumption (especially by the contracting muscle) with an increase of 15-fold in the rate of whole body O_2_ uptake and an increase of more than 100-fold in the O_2_ flux in active muscles [14]. 

Induction of oxidative and nitrosative stress in boys in adapting to physical stress during training and competitive periods was recently studied. Young men who systematically performed muscular work were displayed to have a high content of both markers of different ways to generate superoxide radicals and markers of nitrosative stress [15]. The increase in the degree of adverse effects on the body from intensive training and competitive loads was accompanied by pronounced adaptive changes in the hierarchy of oxidizing constitutive de novo synthesis of nitric oxide, as well as its non-oxide reutilization synthesis (three times higher). Dis-adaptation of the organism of boys at the end of the competition period is reflected in the growing levels of ROS generation (superoxide radical: 3.5 times higher, hydrogen peroxide: 2.5 times higher). The products of purine nucleotides degradation were two times higher, and the increase in the content of the nitrate anion was 2.5 times higher [15]. Other studies during the training period with reports of systemic oxidative stress and induction of muscle damage markers show that high intensity training in healthy non-asthmatic competitive swimmers resulted in marked oxidative stress at the airway and systemic levels, with the hypothesis that oxidative stress may be associated with bronchial hyperresponsiveness, which is often observed during the peak exercise training period [16]. ROS production by contracting muscle during exercise happens through several mechanisms including activation of endothelial xanthine oxidase, electron leakage at the mitochondrial electron transport chain, inflammatory response, increased release, and auto-oxidation of catecholamines. This determines a depletion of cellular antioxidants (such as glutathione) in the blood, and an alteration in the redox balance [12]. Therefore, regular exercise leads to the up-regulation of the body’s antioxidant defense mechanisms, in order to minimize the oxidative stress. ROS and other oxidants enhance oxidative reactions with proteins, lipids, and DNA [17] and this oxidative stress can impair cellular functions determining secondary damage, such as lipo-peroxidation. During exercise, the production of ROS can be higher than the antioxidant capacity of the muscles. As ROS accumulates in the contracting muscles, the oxidation of proteins and lipids can inhibit force production and contributes to the development of acute fatigue [18]. In addition, oxidative DNA modification might inhibit both movement and bactericidal activity of neutrophils, reduce the proliferation of T and B lymphocytes, inhibit natural killer (NK) cells, and damage the cell membrane and other cellular compounds [19]. This negative action of free radicals in lipid, protein, and DNA damage led to the analysis of the efficacy of dietary antioxidant supplementation. Vitamin and mineral supplements are often used by athletes as ergogenic aids for improving performance. 

In this respect, *n*-3 PUFAs could bring various benefits to athletes (Table 1) by attenuating the generation of oxidative stress and, thus, improving muscular performance and immune function. 

Several experimental studies showed that a dietary intake of *n*-3 PUFAs and the improvement in omega-6 and omega-3 ratio could modulate the immune and inflammatory response. After a three-week supplementation with 3.2 g EPA and 2.2 g DHA, an increased content of EPA in neutrophils and monocytes was reported [28]. The anti-inflammatory effects of fish oils are partly mediated by inhibiting the 5-lipoxygenase pathway in neutrophils and monocytes and inhibiting the leukotriene B4 (LTB4)-mediated function of leukotriene B5 (LTB5). In addition, omega-3 decrease interleukins IL-1 and IL-6 inhibits inflammation. For example, rheumatoid arthritis has a strong inflammatory component, which is observed through increased interleukin 1, IL-1 [29]. *N*-3 PUFAs reduce IL-1 as well as the number of swollen and tender joints. Supplementation with EPA and DHA and the dietary change in the *n*-6/*n*-3 ratio appears to be an effective treatment for patients associated with traditional therapies. Similarly, they might be helpful in preventing and contrasting inflammatory joint pain, which can relate to repeated mechanical stress, overuse, and subsequent joint wear, which is typical of sport practice. 

Inflammation is characterized by an increase of prostaglandins (PGs), cytokines, and other pro-inflammatory mediators. The ROS produce peroxidation of phospholipid membranes and damage DNA and intracellular proteins. A diet rich of n-3 PUFAs provides photoprotection and contrasts the risk of skin tumors induced by ultraviolet [30]. They compete with arachidonic acid (AA) for the metabolism by cyclooxygenases (COX)/lipoxygenases, which decrease PGs and cytokines [31]. *N*-3 PUFAs reduce oxidative, inflammatory, and vasogenic processes. In this regard, they were tested in several studies in order to display the reduction of the symptoms of atopic dermatitis, sunburn, aging, and skin infections caused by *P. Acnes* and *S. Aureus* because of their anti-microbial and anti-inflammatory action [32]. A higher consumption of *n*-3 PUFAs improve the response of anti-inflammatory cytokines, LTB3 and PGE3, against the production of AA.

As well as altering eicosanoid production, n-3 PUFAs can also reduce activation of the NF-κB pathway, reducing inflammatory cytokine production contrasting the omega-6 fatty acid AA, which is a known stimulator of NF-κB activity [33]. They also prevent the degradation and subsequent translocation of the NF-κB complex to the nucleus where it induces transcription of inflammatory cytokines. In addiction, a reduction in circulating tumor necrosis factor α (TNF-α) concentrations, as well as in the expression of inflammatory cytokines and cell surface adhesion molecules has been observed [33].

## 3. *N*-3 PUFAs and the Health of Skeletal Muscle

Skeletal muscle performance is usually determined by the use of standard measurements such as the rate of muscle protein synthesis, muscle mass, maximum voluntary contraction, rate of torque development, and the markers of muscle damage.

Researchers saw a positive effect of *n*-3 PUFAs on muscle anabolism and catabolism [24] not only in cancer cachexia [34] but also in healthy volunteers, with a positive impact on the maintenance of muscle. Both in vivo [35] and in vitro [36] studies show a significant increased muscle protein synthesis in both young and older subjects after eight weeks of 4 g *n*-3 PUFAs daily administration [37]. Similarly, six months of supplementation (3.36 g/day) resulted in an increased muscle mass (+3.6%) and strength (+4%) in older people [24]. Another study concerning muscle recovery and soreness after performing eccentric biceps curls displayed that seven days of 3 g/day *n*-3 PUFA supplementation decreased post-exercise muscle damage and soreness [38].

Positive findings in muscle recovery and, subsequently, training adaptation, were reported in other similar studies [39,40,41,42]. *N*-3 PUFAs attenuated the loss of muscle strength and range of motion, blood markers of inflammation such as TNF-α, and markers of muscle damage, such as myoglobin, creatine kinase, and skeletal muscle slow troponin I [43]. In addition, DHA seems to increase lipid oxidation and insulin sensitivity in skeletal muscle and it can stimulate glycolytic capacity in myocytes. *N*-3 PUFAs can probably improve athletic performances, through a modulation on cell membranes’ permeability and on insulin sensitivity, which makes the muscle cells more permeable with regard to necessary nutrients, such as glucose and amino acids [44,45]. This is supported by an up-regulation of the glucose transporter type 4 (GLUT4). Based on these studies, *n*-3 PUFAs appears to be a potent stimulator of metabolism in muscle cells and a potential ergogenic aid [44,45]. An interesting study in older adults showed that *n*-3 PUFAs supplementation augments the hyper-aminoacidemia-hyperinsulinemia induced an increase in the rate of muscle protein synthesis in older adults. Omega-3 fatty acids, therefore, likely attenuate the anabolic resistance and may potentially be useful as a therapeutic agent against catabolic processes.

The exact mechanism acting on the muscle-protein synthesis process is not entirely clear, but it resulted in being partially mediated via increased activation of the mammalian target of the rapamycin (mTOR)-ribosomal protein S6 kinase beta-1 (p70s6k) signaling pathway, which is considered an integral control point for muscle cell growth. This results in an anabolic incitement and induces an increase in the muscle-protein synthesis rate [26]. Therefore, *n*-3 PUFAs likely attenuate the catabolic trend, so that they may be potentially useful as a therapeutic agent to treat sarcopenia and osteoporosis [26]. At the same time, they can be recommended as a good supplement to athletic populations to improve some aspects of recovery during training or in competition. In addition, *n*-3 PUFAs chronic supplementation was demonstrated to enhance neuromuscular activity in animal studies. Some alterations in the membrane composition and fluidity may accelerate conductance of action potentials down the neurons, which increases the motor unit firing rate onto the sarcolemma [46]. The same mechanism could also happen in human skeletal muscle. A 21-days *n*-3 PUFAs supplementation in humans was reported to enhance muscular strength and neuromuscular recruitment following exercise training programs [47] likely because DHA is the essential constituent of neuronal membrane phospholipids and it is fundamental for neuronal pathways.

*N*-3 PUFAs supplementation was also demonstrated to reduce muscle soreness and maintain muscle function following eccentric exercise-induced muscle damage [20]. The effectiveness of consuming a protein-based supplement containing 1546 mg of n-3 PUFA (551 mg EPA and 551 mg DHA) twice daily was compared to a protein-based placebo on muscle soreness, countermovement jump performance, and psychological well-being in 20 professional Rugby Union players for five weeks of pre-season training. Players completed a questionnaire assessing fatigue, sleep, stress, and mood each morning of training. They also performed countermovement jump tests once or twice per week. From day 20, a moderate beneficial effect of the supplement on fatigue was observed. In terms of practical relevance, the moderate beneficial effect of adding fish oil to a protein-based supplement on muscle soreness translated into the better maintenance of explosive power in elite Rugby Union players during pre-season training [20].

## 4. *N*-3 PUFAs and the Availability of Energy

The health of the muscle and also energy metabolism is a crucial point in exercise performance [48]. In human myotubes, 24 h pretreatment with 100 µM EPA increased the suppressibility caused by acute exposure to glucose on fatty acid metabolism and increased the substrate-regulated flexibility of the cells. Furthermore, pretreatment with ALA and DHA increased substrate-regulated flexibility to the same extent as EPA, which suggests a possible favorable effect of n-3 PUFAs on skeletal muscle substrate handling and metabolic switching [21]. This can determine a global improvement in muscle metabolic flexibility, which is the ability to switch from a certain metabolic substrate to another one when necessary [22]. In addition, *n*-3 PUFAs administration can limit the accumulation of the intramyocellular lipid in type I muscle fibers. Therefore, n-3 PUFAs supplementation could be beneficial for endurance athletes, who largely depend on fatty acid as a substrate to sustain prolonged efforts [48]. A potential improvement in endurance performance has been suggested by another study, which found that *n*-3 PUFAs supplementation (1.1 g per day), versus a placebo, resulted in a significant increase in VO_2_-max (+3.7 mL kg^−1^ min^−1^) and in endothelial function [49]. This augmented maximal oxygen uptake in endurance-trained athletes can be related to some beneficial cardiovascular modifications. Furthermore, *n*-3 PUFAs has been found to decrease submaximal and peak heart rate as well as body oxygen consumption during exercise [50], resting heart rate variability [51], submaximal and resting heart rate, systemic vascular resistance, and diastolic blood pressure [52]. Furthermore, in rat hindlimb muscle, *n*-3 PUFAs supplementation also lowered skeletal muscle oxygen consumption during contractions, which provided fatigue resistance and improved contractile recovery in vivo [53].

## 5. Immuno-Stimulating Effect of *n*-3 PUFAs

Physical exercise can exert negative immunomodulatory effects and provide an opportunity for infectious agents to enter the body and cause diseases. Several post-exercise alterations in immune functions have been displayed including augmented pro-inflammatory cytokine production, decreased neutrophil function, and NK cell cytotoxicity [54]. Post-exercise production of IL-6 resulted in a decrease after increasing *n*-3 PUFAs intake and higher activated peripheral blood mononuclear cell proliferation was reported in fish oil supplemented athletes. In particular, a study investigated the effect of supplementation with fish oil on the immune response from an acute bout of endurance exercise. Sixteen male subjects underwent a six-week double blind randomized placebo controlled supplementation trial involving two groups (fish oil or placebo oil, 3 g/day). They attended two visits in which the first involved a maximal exercise test and the second involved a 1-h bout of endurance exercise on a cycle ergometer at 70% VO_2_. Blood samples were taken pre-supplementation, pre-exercise (post-supplementation), immediately, 1 h and 3 h post-exercise, which demonstrates that fish oil supplementation reduces increases in peripheral blood mononuclear cells (PBMC) IL-2 production [55]. After a single bout of high-intensity or long-duration endurance exercise, such as running, there is clear evidence of immunosuppression. Therefore, athletes are more prone with the development of upper respiratory tract infections [20], which can interfere with their performance in both training and competition. Many studies displayed that n-3 PUFAs supplementation could ameliorate immune functions after exercise and contrast the incidence of upper respiratory tract infections [56,57]. For example, a supplementation (1.8 g/day) for six weeks before a major competition reduced PGE2 levels and increased interferon-gamma (IFN-γ) production [20,58]. A similar supplementation (1.6 g/day for six weeks) improved the exercise economy and reduced the perceived exertion during sub-maximal steady-state exercise in normal, healthy, untrained men too. It resulted in an increase in IL-2 production from peripheral blood mononuclear cells and in NK cell activity [59]. Dietary intake of EPA and DHA leads to increased incorporation of these fatty acids into cell membranes, which partly replaces arachidonic acid. Future targeted lipidomics-based studies will help discover whether *n*-3 PUFAs supplementation actually enhances inflammation resolution in athletes after exercise [60]. Larger and longer duration studies are needed to measure the potential benefits of *n*-3 PUFAs supplementation in athletic groups, with more careful consideration given to the inflammatory outcomes and the use of targeted lipidomics procedures. 

## 6. *N*-3 PUFAs and Cardiovascular Health: Anti-Arrhythmic Potential and Vasodilatation

Clinical evidence suggests that EPA and DHA help reduce cardiovascular risk factors, such as high cholesterol and high blood pressure. Fish oil has been shown to lower levels of triglycerides, and to lower the risk of cardiovascular death and abnormal heart rhythms. It can prevent ventricular arrhythmias that lead to sudden death [61,62,63]. Sudden death is a crucial topic in sport and it is conventionally defined as an unexpected and instantaneous death occurring during or immediately after (i.e., within 1–3 h) exercise, due to any cause except violence [64]. Because more than 50% of all sudden deaths from cardiac causes occur in people with no history of cardiac disease [63], preventive efforts must address this segment of the population to have a substantial effect on the overall incidence of sudden death from cardiac causes. Prospective data from observational studies and randomized trials suggest that the long-chain *n*–3 PUFAs found in fish may reduce the risk of sudden death from cardiac causes, not only among unhealthy subjects, such as patients starting hemodialysis or with atrial fibrillation [65,66], but also among men without a history of cardiovascular disease [63]. This beneficial effect on the risk of sudden death from cardiac causes in observational studies and randomized trials could be due in part to the anti-arrhythmic effects of *n*–3 PUFAs, as reported from experimental models [67,68].

The mechanisms explaining these anti-arrhythmic effects include modulation of sodium, potassium, and L-type calcium channels [69,70], the inhibition of thromboxane production [71,72], and the beneficial effects on heart-rate variability [73,74]. N-3 PUFAs were demonstrated to make heart cells less excitable (by modulating ionic channels) and also to slower atrioventricular conduction and substantially lower the probability of having a prolonged QT interval [75]. Other indirect effects of long-chain n–3 fatty acids include lowering concentration (in both plasma and cell membranes) of the non-esterified fatty-acids, which have multiple pro-arrhythmic properties [76] and have recently been associated with an increased risk of sudden death among men enrolled in the Paris Prospective Study [77]. Their anti-inflammatory action could be the key to further cardiovascular beneficial effects. *N*-3 PUFAs decrease pro-inflammatory eicosanoid mediators’ production from arachidonic acid. On the other side, they increase the production of anti-inflammatory eicosanoids from EPA. They decrease both chemotactic responses of leukocytes and adhesion of molecule expression on leukocytes and on endothelial cells. They also decrease intercellular adhesive interactions. Together, these anti-inflammatory actions may contribute to omega-3 anti-atherogenic effects [63,78], so that fish oil also appears to help prevent and treat atherosclerosis by slowing the development of plaque and blood clots in arteries. Another study in a Japanese population found that high intake of fish was inversely associated with death caused by intracerebral hemorrhaging [79]. EPA and DHA can influence membrane fluidity, interact with Peroxisome Proliferator-Activated Receptors (PPARs) and other transcription factors and sterol regulatory element binding proteins, and are substrates for enzymes such as COX, lipoxygenase, and cytochrome P450 [80]. As a result, *n*-3 PUFAs can induce haemodynamic changes, improve endothelial function and arterial compliance, decrease arrhythmias risk, and inhibit inflammatory pathways. Strong evidence suggests that DHA is more efficient in decreasing blood pressure, heart rate, platelet aggregation, and improving both the endothelial function and the ratio between HDL and LDL cholesterol compared to EPA [80]. These work strongly supports the role of omega-3 in decreasing total cardiovascular risk and mortality, so that their daily supplementation is highly recommended for both primary and secondary cardiovascular prevention.

## 7. *N*-3 PUFAs and Inflammatory Diseases

### 7.1. Role of n-3 PUFAs in Asthma and Exercise-Induced Bronchoconstriction

Asthma is a chronic inflammatory respiratory disorder, characterized by bronchial constriction with coughing, wheezing, and breathlessness, whose risk is increased by vigorous physical exercise [81]. A recent study defined a specific phenotype of asthma in elite athletes known as the so-called “sport asthma” defined by respiratory symptoms and bronchial hyperresponsiveness without allergic features [82]. In fact, this airway hyperresponsiveness was higher among elite athletes for many possible reasons. Athletes in endurance sports that requires intense breathing, such as skiing and long distance running or cycling [83], are particularly prone to asthma development likely due to high volume ventilation of dry air [84]. Asthma prevalence is also high in competitive swimmers, due not only to high-volume ventilation but also to inhalation of chlorine derivatives in the swimming pool [85].

Hyperpnoea with cold dry air is a noticeable environmental stress to airways that determine parasympathetic stimulation of airways, which contributes to exercise-induced bronchoconstriction. In fact, during the winter season, cross-country skiers develop signs of phlogosis, such as bronchial deposition of tenascin and lymphoid follicles [86]. Mechanisms for bronchial obstruction in the sport population include the osmotic and the thermal hypotheses, but, recently, the presence of epithelial injury and inflammation in the airways of athletes was demonstrated. In addition, neuronal activation was suggested as a potential modulator of bronchoconstriction [86].

Epidemiological studies suggest that dietary n-3 PUFAs may have beneficial effects on asthma because of their anti-inflammatory mechanisms of action [87]. The low incidence of asthma in Eskimos could be derived from their great intake of omega-3 fat fish [88]. A reduction of bronchial inflammation due to an omega-3 dietary supplementation was repeatedly reported [89]. A three-week daily supplementation with 3.2 g of EPA and 2.0 g of DHA reduced eicosanoids and pro-inflammatory cytokines concentration in the sputum of asthmatic patients [25]. Another study compared the effects of a widely used anti-LT medication and daily omega-3 supplementation with 3.2 g EPA+ 2.0 g DHA in asthmatic patients for three weeks. The study demonstrated that both fish oil and the anti-LT medication were independently effective in attenuating airway inflammation and hyperpnoea-induced bronchoconstriction [90]. Similarly, six weeks of dietary supplementation with 120 mg/day of *n*-3 PUFAs comported a significant improvement in infant bronchial asthma’s lung function [91]. Specifically, *n*-3 PUFAs were helpful against exercise-induced bronchoconstriction [25], which is an important limiting factor in athletic performance [92]. In this respect, the effects of 5.2 g/day *n*-3 PUFAs supplementation versus the placebo was valued in asthmatics with the usual exercise-induced bronchoconstriction. Post-exercise lung function improved the concentration of sputum immune cells while pro-inflammatory eicosanoids and cytokines (IL-1β and TNF-α) decreased.

### 7.2. Role of n-3 PUFAs in Osteoarthritis and Joint Pain

Joint pain is extremely common in the athletic population due to local biomechanical factors, such as the degree of joint loading and abnormal load, as well as the frequent occurrence of joint injuries. In particular, athletes participating in sport with joint torsional movements (tennis, football, volleyball, and alpine skiing) or incremented articular impact loading (football, basketball, running, and handball) are more at risk of articular cartilage damage [93]. In addition, the occurrence of early osteoarthritis in athletes has been frequently described, especially in sports including rapid acceleration/instantaneous deceleration or continuous high impact on joints [94]. Not only high levels of impacts and repeated torsional loading, but also all cartilage, ligamentous, and subchondral bone lesions may lead to the development of post-traumatic osteoarthritis in sport [95]. However, cartilage health and tolerance play a crucial role in the development of joint degeneration. In this respect, *n*-3 PUFAs could be helpful in promoting and maintaining joint health. Joint pain is often caused by infiltration of inflammatory cells into the synovium, with secretion of cytokines, eicosanoids, and other phlogosis mediators [96].

Under inflammatory conditions, AA is released from the membrane phospholipids, COX increased their activity and this resulted in the formation of pro-inflammatory lipid mediators such as tromboxanes (TX) and PG. In particular, PGE2 increases vasodilatation, vascular permeability, and the release of destructive matrix metalloproteinases, which leads to further tissue damage, edema, and pain [97].

Fish oil supplementation (3 g/day) reduced both incidence, from 93% to 69%, and the severity (mean peak severity score passed from 9.8 to 6.7) of type II collagen-induced arthritis. In addition, arthritis onset was delayed in mice from 25 days to 34 days [98].

Similarly, a New Zealand green lipped mussel *Perna canaliculus* representing another potential source of *n*-3 PUFAs was reported to reduce inflammation in both animal studies and patient trials with an 89% decrease in pain symptoms and a 91% improved quality of life. A 12-week supplementation of 400 mg PCSO-524™ (a non polar lipid extract from *Perna canaliculus*) achieved the same anti-inflammatory benefits as 1200 mg of standardized fish oil, which resulted in reduced joint stiffness and pain, increased grip strength, and an improved walking pace in a small group of people with osteoarthritis. Furthermore, this enabled a reduction of analgesics intake [99].

## 8. Potential Adverse Effects of *n*-3 PUFAs

Unfortunately, not only medications but also dietary supplements or nutraceuticals could lead to adverse effects. Despite the benefits listed above, there are potential risks associated with excessive usage of *n*-3 PUFAs. Important potential adverse effects include altered platelet function. The presence of EPA and DHA leads to the production of thromboxane A_3_, which is a less potent platelet activator than thromboxane A_2_. Supplementation of EPA and DHA, therefore, may affect platelet activation because of the different eicosanoids produced, which leads to an antithrombotic effect that causes detrimental effects for wound healing [100]. The potential for adverse effects on wound healing may be at its greatest immediately after trauma or surgery. The effect on wound healing likely depends on the amount and the duration of supplementation, and the severity of the wound. Another side effect can be represented by lipid peroxidation. Lipid peroxidation is characterized by a free radical attack on an unsaturated fatty acid and can occur in the presence of oxygen. Long-chain, highly unsaturated fatty acids such as EPA and DHA, which accumulate in cell membranes, are at high risk of peroxidation. If antioxidants are not provided at adequate concentrations, membrane phospholipid fatty acids can be vulnerable to peroxidation and free radicals can form as a result. Lipid peroxidation can be detrimental because of effects on the stability of cell membranes and also as a result of free radical attacks on proteins and DNA [101]. The effects of lipid peroxidation can be avoided by supplementing diets enriched in *n*-3 PUFAs with antioxidants. Further problems may come from toxin exposure (given the potential presence not only of useful marine bioactives [102,103,104] but also of sea contaminants) and nutrient-drug interactions. In humans, the interaction with simvastatin was displayed and a decrease in its blood lipid concentrations was observed [105]. For other medication, nutrient-drug interactions may exacerbate adverse effects that can occur with *n*-3 PUFAs supplementation alone. *N*-3 PUFAs supplementation is contra-indicated during antiplatelet and anticoagulant treatment because of the synergistic effect on bleeding times when administered together [106].

Clinicians should understand the adverse effects that may occur with *n*-3 PUFAs supplementation, and that potential risks should be assessed in conjunction with the potential benefits. Adverse effects are likely to be dose-dependent. It is necessary to understand the necessary dosages and which dietary concentration to aim for, when recommending *n*-3 PUFAs supplementation.

## 9. Conclusions

Athletes experience regular cycles of physiological stress accompanied by transient inflammation, oxidative stress, and immune perturbations. Exercise activates multiple molecular and biochemical pathways with many involving the immune system, and there are increasing data points indicating that these are sensitive to nutritional influences. Nutritional support has the potential to partially mitigate these exercise-induced changes without interfering with signaling activities that are needed for training adaptations. The most effective nutritional countermeasures for athletes include *n*-3-PUFAs, bovine colostrum, terrestrial supplements, and antioxidants, such as vitamins, carotenoids, poliphenols [107,108,109,110], probiotics, β-glucans [60] and nutraceuticals [111]. *N*-3 PUFAs seem to be among the most useful supplements for a huge range of the population (premature infants, elderly with sarcopenia, athletes, and patients with metabolic and inflammatory diseases). Since only the Eskimos, the Japanese, and a few other small groups of people do not require these supplements, these fats should be added to foods, rather than be used solely as food supplements. Furthermore, *N*-3 PUFAs maintain their properties when packaged in wholesome foods other than fish. Concurrently, omega-6 dietary intake reduction is required, in order to reduce the omega-6/omega-3 ratio to the extent provided by the evolution of human biology. There is good evidence from murine and human studies regarding the Paleolithic diet, the diet of Crete, and the Okinawa diet that the physiological *n*-6: *n*-3 ratio should be 1:1 or 2:1. Japan has already recommended a ratio of 2:1. Nutritional strategies and supplements such as *n*-3 PUFAs can result in optimal training gains, enhanced recovery, reduced risk of illness, and a high-level competition performance. In addition, it was demonstrated that they could affect mood and emotional states. Supplementing 24 female elite soccer players with 3.5 g per day of DHA-rich fish oil for four weeks produced perceptual-motor benefits (i.e., improvements in complex reaction time and efficiency). This supports the view that DHA may improve performance in sports where perceptual-motor activity and decision-making are the keys to success [112]. If the observed positive associations are causal, increasing the intake of *n*–3 PUFAs by eating more fish or by taking supplements is an intervention that could be applied to this segment of the population. This has the potential to be an ergogenic aid that enhances training and sport performance at low cost and little risk.

## Figures and Tables

**Table 1:** Summary of the impact of *n*-3 PUFAs in athletes.

Protocol	Key Results
551 mg eicosapentaenoic acid (EPA) and 551 mg docosahexaenoic acid (DHA) twice daily, during five weeks of pre-season rugby training	Reduced fatigue in countermovement jump tests [20]
24-h exposure with 100 microM EPA in human myotubes	Augmented adaptability and upregulation of specific genes implicated in fatty acid beta-oxidation with global improvement in muscle metabolic flexibility [21]
Four-week supplementation with *n*-3 PUFAs 1.1 g per day	Significant increase in maximal oxygen uptake (VO_2_-max) and in endothelial function [22]
14-days diet enriched with 5% cod liver oil followed by 14 days immobilization	Reduced myosin heavy chain loss during 14 days of hind limb immobilization [23]
Six-months supplementation with 1.8 g EPA, 1.5 g DHA daily	Increased hand grip and muscle strength [24]
Three-week supplementation with 3.2 g of EPA and 2.0 g of DHA	Reduced eicosanoids and pro-inflammatory cytokines concentration in the sputum of asthmatic athletes [25]
Six-months supplementation with 3.36 g/day of *n*-3 PUFAs	Increased muscle mass and strength in older people [24]
Eight-weeks supplementation with 1.86 g EPA, 1.5 g DHA daily	Augmented muscle protein synthesis, enhanced rapamycin (mTOR)-ribosomal protein S6 kinase beta-1 (p70s6k1) signaling after hyperaminoacidemic-hyperinsulinemic clamp [26]
Supplementation with 0.4 g EPA, 0.3 g DHA (60 days pre-training and 90 days during training)	Potential training increase in peak torque and rate of torque development (Knee extensor, flexor, plantar, and dorsiflexor) [27]
